# Childhood violence experience interacts with BDNF Val158Met polymorphism and modify internet addiction risk

**DOI:** 10.1192/j.eurpsy.2022.351

**Published:** 2022-09-01

**Authors:** N. Chuprova, T. Merkulova, M. Solovieva, A. Nikolishin, A. Trusova, S. Grechany, V. Soldatkin, A. Yakovlev, P. Ponizovsky, R. Ilyuk, A. Egorov, E. Krupitsky, A. Kibitov

**Affiliations:** 1 Serbsky National Medical Research Center on Psychiatry and Addictions, Molecular Genetics Laboratory, Moscow, Russian Federation; 2 Serbsky National Medical Research Center on Psychiatry and Addictions, Laboratory Of Molecular Genetics, Moscow, Russian Federation; 3 Federal State Budgetary Istitution Serbsky National Medical Research Centre for Psychiatry and Narcology of Ministry of Heath of the Russian Federation, Laboratory Of Molecular Genetics, Moscow, Russian Federation; 4 Saint Petersburg State University, Department Of Medical Psychology And Psychophysiology, Saint Petersburg, Russian Federation; 5 Saint Petersburg State Pediatric Medical University, Department Of Psychiatry And Addictology, Saint Petersburg, Russian Federation; 6 Rostov State Medical University, Department Of Psychiatry, Addictology And Medical Psychology, Rostov-on-Don, Russian Federation; 7 Lipetsk Regional Addiction Hospital, Department Of Addictology, Lipetsk, Russian Federation; 8 Serbsky National Medical Research Center on Psychiatry and Addictions, Department Of Therapy Of Mental Disorders Complicated By Addiction Diseases, Moscow, Russian Federation; 9 Bekhterev National Medical Research Center for Psychiatry and Neurology, Department Of Addictology, Saint Petersburg, Russian Federation; 10 Sechenov Institute of Evolutionary Physiology and Biochemistry of the Russian Academy of Sciences, Laboratory Of Neurophysiology And Pathology Of Behavior, St.Petersburg, Russian Federation

**Keywords:** BDNF Val158Met, internet addiction, adverse childhood experiences

## Abstract

**Introduction:**

Internet-addiction (IA) is one of the most common non-chemical (or behavioral) addictions with genetic impact and substantial effects of psychological and personality characteristics, taking into account the childhood traumatic experience. Gene-environment interactions (GxE) may substantially impact on the risk of Internet-addiction (IA).

**Objectives:**

Aim: to test the associations between the functional polymorphism rs6265 (Val66Met) in brain-derived neurotrophic factor (BDNF) gene, affecting BDNF function, and childhood traumatic experience and their GxE interactions with IA risk.

**Methods:**

In total 456 participants were screened with Chinese Internet Addiction Scale (CIAS) to cut a cohort on two groups: IA (CIAS total score ≥ 65, n=100) and controls (CIAS total score less 64, n=356). The Adverse Childhood Experiences International Questionnaire (ACE-IQ) was used to assess childhood traumatic experience using its main domains: parents (P), family (F), abuse (A) and violence (V). BDNF Val158Met polymorphism was detected by RT-PCR.

**Results:**

Logistic regression revealed associations of P scores with increased IA risk only after adjustment for sex and age (p=0.01, OR=1.166, 95%CI[1.038-1.309]) and V scores with decreased IA risk (p=0.000, OR=0.799, 95%CI [0,233;0,744] only before adjustment. No associations of F and A with IA risk were found. BDNF Val158Met per se was not associated with IA risk, but significant effect of interaction V score*BDNF rs6265 CC on IA risk in “protective” manner was revealed (р=0.039, OR=0.873, 95%CI[0.768-0.993]) in a model adjusted for sex and age.

**Conclusions:**

Childhood violence experience interacts with BDNF Val158Met polymorphism and CC (ValVal) genotype may be possibly protective factor decreasing the internet addiction risk

**Disclosure:**

This work was carried out with the financial support of the Russian Foundation for Basic Research: RFBR grant # 18-29-22079

